# Telocytes: a potential defender in the spleen of Npc1 mutant mice

**DOI:** 10.1111/jcmm.13024

**Published:** 2016-11-18

**Authors:** Bichao Zhang, Ciqing Yang, Liang Qiao, Qiuling Li, Congrui Wang, Xin Yan, Juntang Lin

**Affiliations:** ^1^ College of Life Science and TechnologyHenan Key Laboratory of Medical Tissue RegenerationXinxiang Medical UniversityXinxiangChina; ^2^ College of Biomedical EngineeringStem Cell and Biotherapy Engineering Research Center of HenanXinxiang Medical UniversityXinxiangChina; ^3^ Institute of Anatomy IUniversity of Jena School of MedicineJena University HospitalJenaGermany

**Keywords:** telocytes, spleen, Niemann–Pick disease, type C1, hematopoietic stem cells, macrophages

## Abstract

Niemann–Pick disease, type C1 (Npc1), is an atypical lysosomal storage disorder caused by autosomal recessive inheritance of mutations in *Npc1* gene. In the Npc1 mutant mice (Npc1^−/−^), the initial manifestation is enlarged spleen, concomitant with free cholesterol accumulation. Telocytes (TCs), a novel type of interstitial cell, exist in a variety of tissues including spleen, presumably thought to be involved in many biological processes such as nursing stem cells and recruiting inflammatory cells. In this study, we found that the spleen is significantly enlarged in Npc1^−/−^ mice, and the results from transmission electron microscopy examination and immunostaining using three different TCs markers, c‐Kit, CD34 and Vimentin revealed significantly increased splenic TCs in Npc1^−/−^ mice. Furthermore, hematopoietic stem cells and macrophages were also elevated in Npc1^−/−^ spleen. Taken together, our data indicate that splenic TCs might alleviate the progress of splenic malfunction *via* recruiting hematopoietic stem cells and macrophages.

## Introduction

Npc is a cellular lipid trafficking disorder induced by mutation of *Npc1* or *Npc2* genes 1, 2, 3, 4. Npc1 mutations are responsible for 95% NPC patients and Npc2 mutations for 5% 3. The mutation of Npc1 protein disrupts intracellular lipid transport and leads a progressive accumulation of lipids in the late endosomes and lysosomes 5, 6. In Npc1^−/−^ mice, altered metabolism of cholesterol and glycolipid has been found in many tissues and organs, including brain, spinal cord and liver 7, 8, 9, 10, 11, 12. Interestingly, as one of hallmarks of this disease, the enlarged spleen has been identified in both Npc1 patient and mutant mice model 13, 14. The further histological experiment revealed the devastated morphological structures in the Npc1^−/−^ spleen associated with accumulation of cholesterol and lipids and increased activity of macrophages 15.

TCs, a distinct type of interstitial cells, have been described in a broad range of tissues and organs including spleen 16, 17, 18, 19. TC is characterized by a small cell body and several extremely long and thin telopodes (Tps), which form three‐dimensional network and maintain tissue homeostasis 20, 21. TCs can be identified by some specific markers, such as CD34, Vimentin, c‐Kit, PDGFR‐α and‐β 18, 19, 20, 22, 23. Recently, accumulating evidence demonstrated that TCs occupy a strategic position in relation to stem cell niches 24, 25, 26, 27 and Tps also establish contacts with other cells such as lymphocytes, eosinophils, plasma cells or macrophages 28, which suggests them as key players in regeneration and repair of tissues 29, 30. However, how splenic TCs response to Npc1^−/−^ spleen is still unknown, although it has been reported to participate in some pathologic processes 31, 32, 33, 34.

In this study, we found that the size and weight of spleen are significantly enlarged in Npc1^−/−^ mice and that the number of splenic TCs is dramatically increased with double‐immunofluorescence staining for c‐Kit/CD34, Vimentin/CD34 and Vimentin/c‐Kit, which may simultaneously recruit stem cell or macrophage to the nidus. These data indicate that splenic TCs could play a vital role in the pathologic process of Npc1.

## Materials and methods

### Mice and tissue preparation

Male wild‐type (WT) and Npc1^−/−^ mice aged 40 days were housed with a 12‐h light/dark cycle (lights on from 07:00 to 19:00) at constant temperature (25°C). All animal protocols were conducted under the guidelines of The Ministry of Science and Technology of the People's Republic of China [(2006)398] and approved by the Animal Care Committee of Xinxiang Medical University (No. 030032). All mouse strains were kept in a Balb/c background.

Dissected 40d WT and Npc1^−/−^ spleens were fixed overnight in 4% paraformaldehyde which was dissolved in PBS salt solution (PBS, pH = 7.4). The spleens were put in a weigh boat and covered with melted 5% PBS low‐melt agarose (Biowest agarose) at 40°C and allowed to solidify on ice for vibratome section (Leica VT1200s, Wetzlar, Germany).

### Transmission electron microscopy

Dissected 40d WT and Npc1^−/−^ spleens were cut into small pieces of 1 mm^3^ and fixed by 2.5% glutaraldehyde solution (Leagene, Beijing, China) overnight at 4°C. Subsequently, these tissues were washed in phosphate buffer for four times followed by post‐fixation with 1% osmium tetroxide in 0.1 M phosphate buffer for 2 hrs at 4°C. After that, tissues were dehydrated through graded alcohols (50, 70, 90 and 100%) for 30 min each and embedded in Epon 812. Semi‐thin sections were cut at 1.5‐μm by Leica Ultracut R (Solms, Germany) and stained with toluidine blue, and histologically analysed by light microscopy. Ultrathin sections were cut at 70 nm and contrasted with uranyl acetate and lead citrate, and they were examined with an H‐7500 electron microscope (Hitachi, Tokyo, Japan).

### Immunofluorescent staining

Using a Leica VT1200s vibratome, 30‐μm‐thick uniform vertical sections were cut, transferred to 12‐well cell culture plate (Thermo Fisher Scientific, MA, USA) and then were post‐fixed with 4% paraformaldehyde dissolved in PBS (pH = 7.4) for at least 15 min. After washed with PBS for three times, sections were immersed in 0.1% Triton X‐100 for 5 min to penetrate the cytomembrane. After that, sections were immediately blocked with 10% goat serum for 1 hr).

For TCs staining, sections were incubated overnight at 4°C with rabbit monoclonal anti‐CD34 (Abcam, Cambridge, UK) and mouse monoclonal to Vimentin (Abcam), both with the dilution of 1:500 in first antibody diluent (2% goat serum+4% bovine serum albumin+0.3% Triton X‐100 + 0.1% NaN_3_). On the second day, the sections were washed by PBS three times and then incubated with goat anti‐rabbit labelled with Cy3 (Beyotime, Shanghai, China) and goat antimouse labelled with FITC secondary antibodies (Beyotime) diluted 1:500 in secondary antibody diluent (2% goat serum+4% bovine serum albumin +0.1% NaN_3_) at 4°C for 6 hrs. After three times washing in PBS, sections were stained with DAPI (Boster, China) for 10 min and then they were collected with a fine brush and mounted on adhesion microscope slides (CITOGLAS, Jiangsu, China) for microscopy. The same protocol was used for double‐immunofluorescent stainings for CD34/c‐Kit (mouse monoclonal anti‐c‐Kit, 1:500; Santa Cruz biotech, TX, USA) or c‐Kit/Vimentin.

For hematopoietic stem cell staining, double‐immunofluorescent staining for c‐Kit/Nanog (rabbit monoclonal anti‐Nanog, 1:500; Santa Cruz biotech) was preformed and the specific procedure was similar with TCs staining.

For TCs and hematopoietic stem cell or TCs and macrophages costaining, double immunostaining for Vimentin/Nanog or CD34/CD68 (rat polyclonal anti‐CD68, 1:500; Santa Cruz biotech) was performed and the specific procedure was similar with TCs staining.

Using confocal laser scanning microscope (SP8; Leica Microsystems, Wetzlar, Germany), more than 20 images (400× or 40×) were acquired from the central area of splenic sections and merged by LAS AF Lite software (Leica Microsystems).

Counting was performed by two independent observers blinded to the sample classification. The average TC number in these 20 images was expressed as the density of TCs. Using Quantity One software (Bio‐Rad, CA, USA), the images of macrophage staining were scanned to acquire the grey value and the relative grey value of WT and Npc1^−/−^ was considered as the amount of macrophages.

### Statistical analysis

All data were presented as mean ± S.E.M. An independent Student's *t*‐test was applied using SPSS 19.0 software (IBM, Armonk, NY, USA). *P* < 0.05 was considered as statistically significant difference.

## Results

### Enlarged spleen in Npc1^−/−^ mice

**Figure 1:**
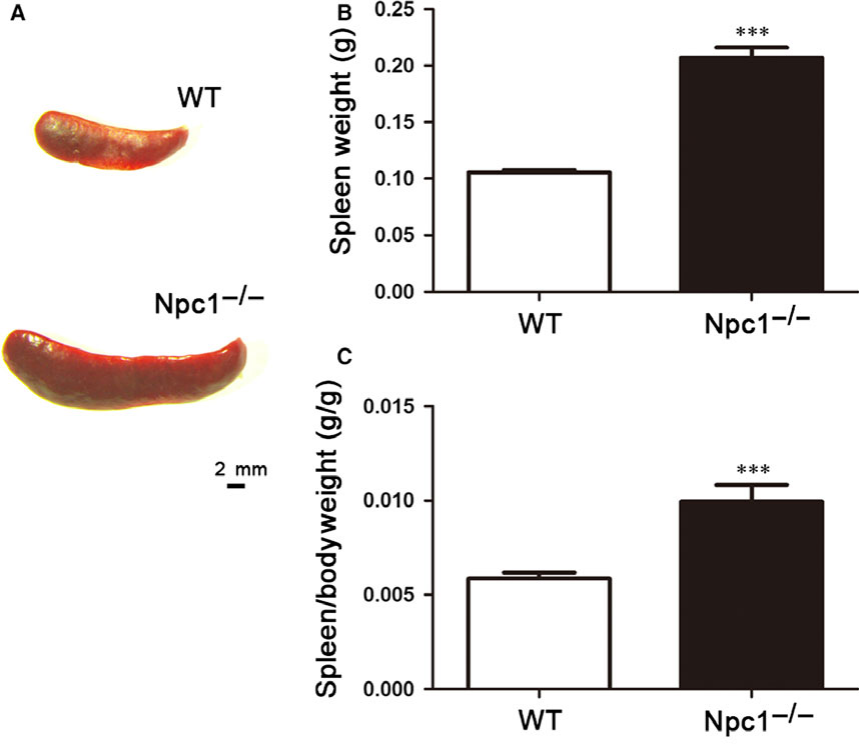
Enlarged spleen in Npc‐1^−/−^ mice. Representative images of general view of the spleen (**A**), spleen weight (**B**) and spleen/bodyweight ratio (**C**). Scale bar = 2 mm labelled in **A**,* n* = 14. ***P < 0.001.

Spleen is thought to be the largest immune organ in organisms, which contains numerous lymphocytes and macrophages. It has been reported that one of hallmarks of Npc is the enlarged spleen 14. Consistent with this, our results confirmed that the spleen appears significantly enlarged in Npc1^−/−^ mice (Fig. 1). In our study, we found that the spleen of Npc1^−/−^ mice is twice as long as that of the wild‐type control, as shown in Figure 1A. In addition, there is a considerable difference in absolute weight between WT and Npc1^−/−^ spleen, almost up to twofold (Fig. 1B). Interestingly, the ratio of spleen/bodyweight is remarkably increased in Npc1^−/−^ mice, compared to the control (Fig. 1C).

### Increased number of splenic TCs in Npc1^−/−^ spleen

**Figure 2:**
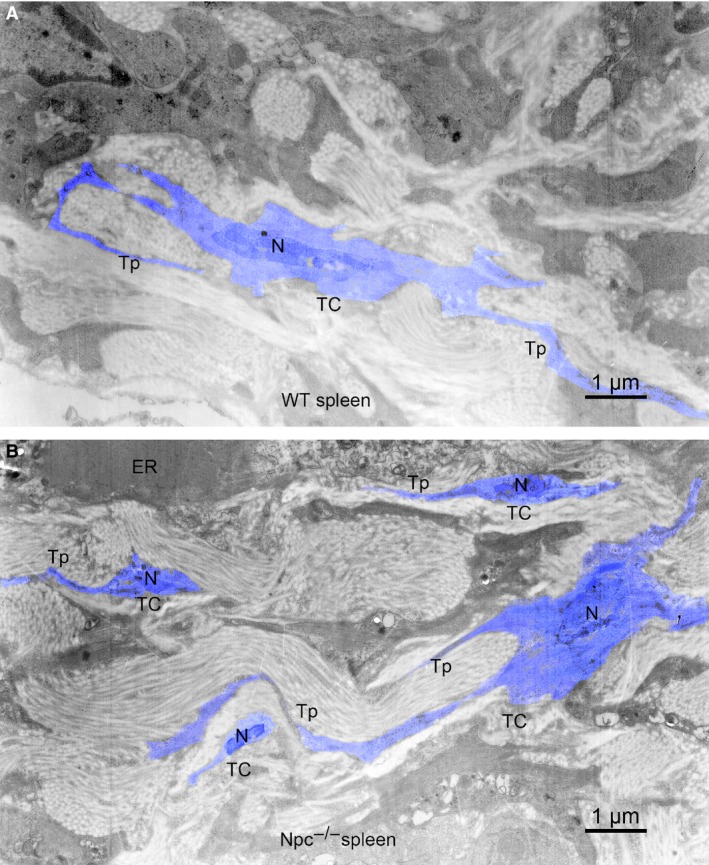
Electron microscope images show that the amount of telocytes (TCs) in the Npc1^−/−^ mice is increased. (**A**) Telocytes (TCs) with telopodes (Tps) in the spleen of WT mice. (**B**) Telocytes (TCs) with telopodes (Tps) in the spleen of Npc1^−/−^ mice; ER, endoplasmic reticulum. Scale bar = 1 μm.

As a golden standard for the identification of TCs, transmission electron microscopy examination has demonstrated the existence of TCs in many tissues and organs, including spleen. As shown in Figure 2, TCs were present in the spleen of WT and Npc1^−/−^ mice and had the distinctive ultrastructural features: an oval or triangular‐shaped cell body and 2 or 3 long and thin Tps, which is consistent with other reports. Furthermore, it is interesting in our study that the splenic TCs are significantly increased in Npc1^−/−^ mice (Fig. 2B) compared to the wild‐type (Fig. 2A). To make it more convinced, three distinctive double immunostainings were performed in the spleen of WT and Npc1^−/−^ mice.

**Figure 3:**
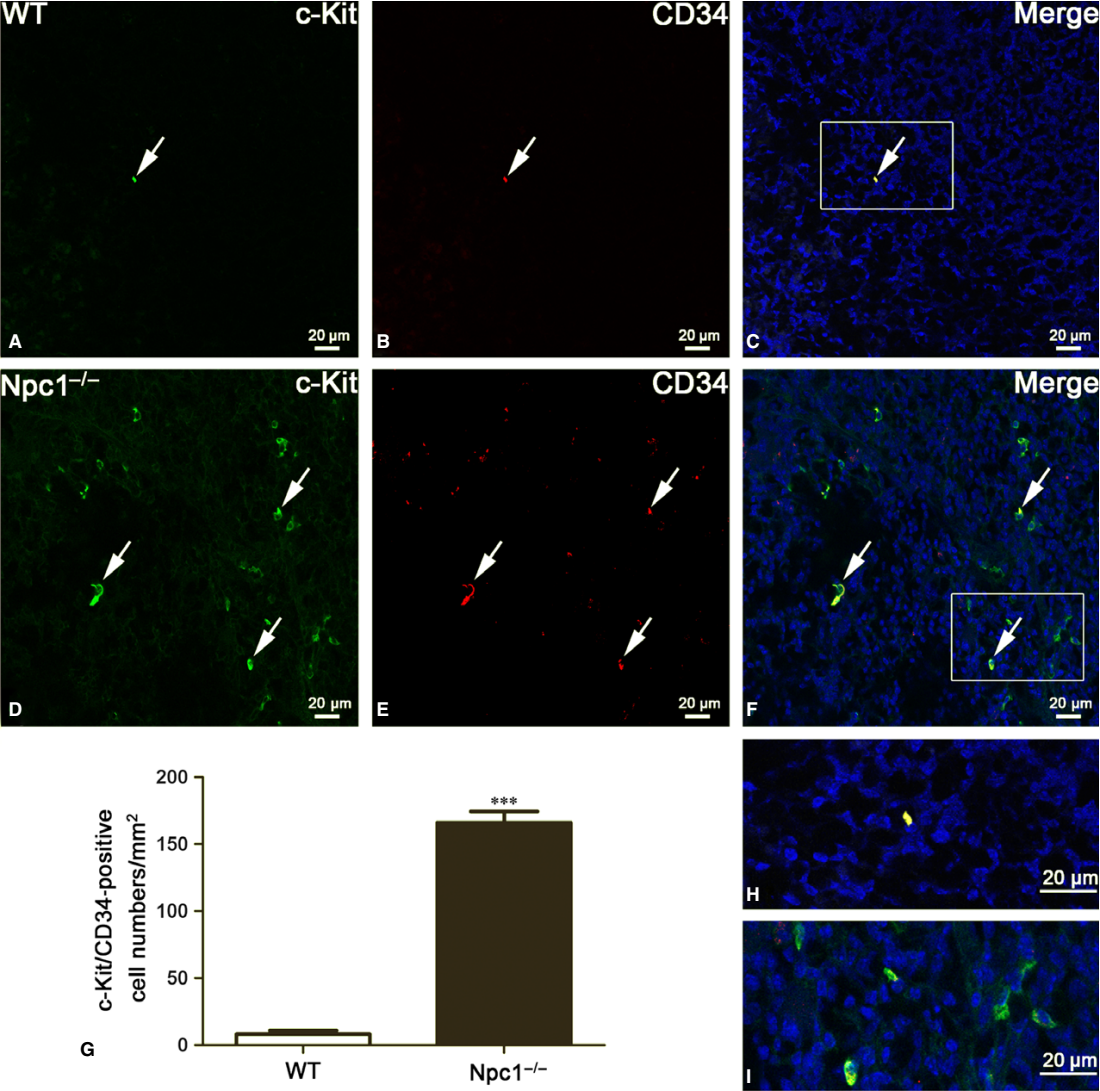
Splenic telocytes (TCs) are identified by c‐Kit/CD34 double‐immunofluorescent staining from confocal laser scanning microscopy. c‐Kit and CD34 double immunostaining show c‐Kit (green in **A** and **D**), CD34 (red in **B** and **E**) and their coexpression merge (yellow in **C** and **F**) in telocytes of WT and Npc1^−/−^ mice. TCs in the spleen of Npc1^−/−^ mice are increased as determined by double‐immunofluorescence labelling for c‐Kit/CD34 (**G**). **H** and **I** are the partial amplification from boxed area in **C** and **F**, respectively. Scale bar = 20 μm labelled in each panel, *n* = 4. ***P < 0.001.

**Figure 4:**
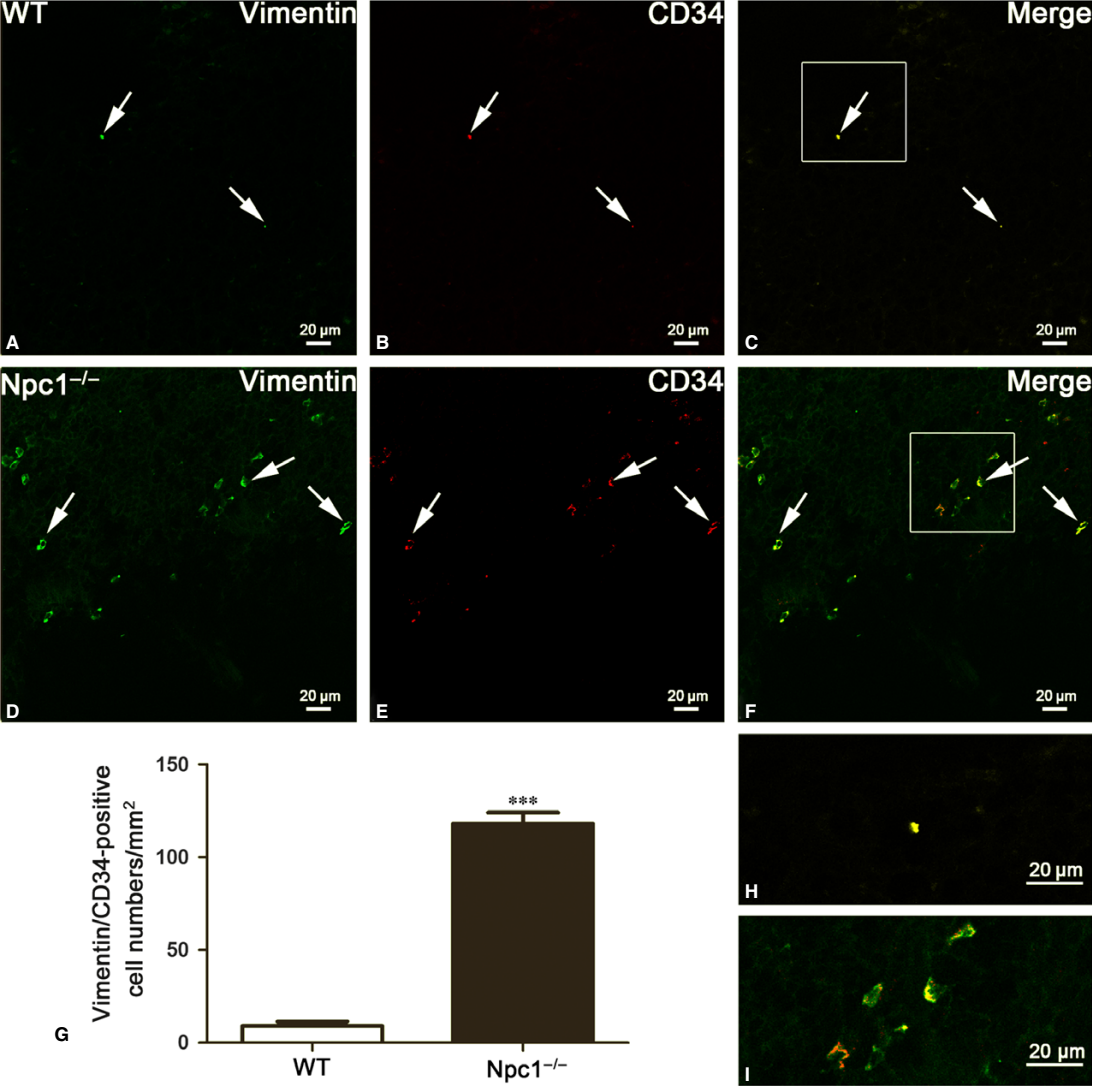
Splenic telocytes (TCs) are identified by Vimentin/CD34 double‐immunofluorescent staining from confocal laser scanning microscopy. Vimentin and CD34 double immunostaining show Vimentin (green in **A** and **D**), CD34 (red in **B** and **E**) and their coexpression merge (yellow in **C** and **F**) in telocytes of WT and Npc1^−/−^ mice. TCs in the spleen of Npc1^−/−^ mice are increased as determined by double‐immunofluorescence labelling for Vimentin/CD34 (**G**). **H** and **I** are the partial amplification from boxed area in **C** and **F**, respectively. Scale bar = 20 μm labelled in each panel, *n* = 4. ***P < 0.001.

**Figure 5:**
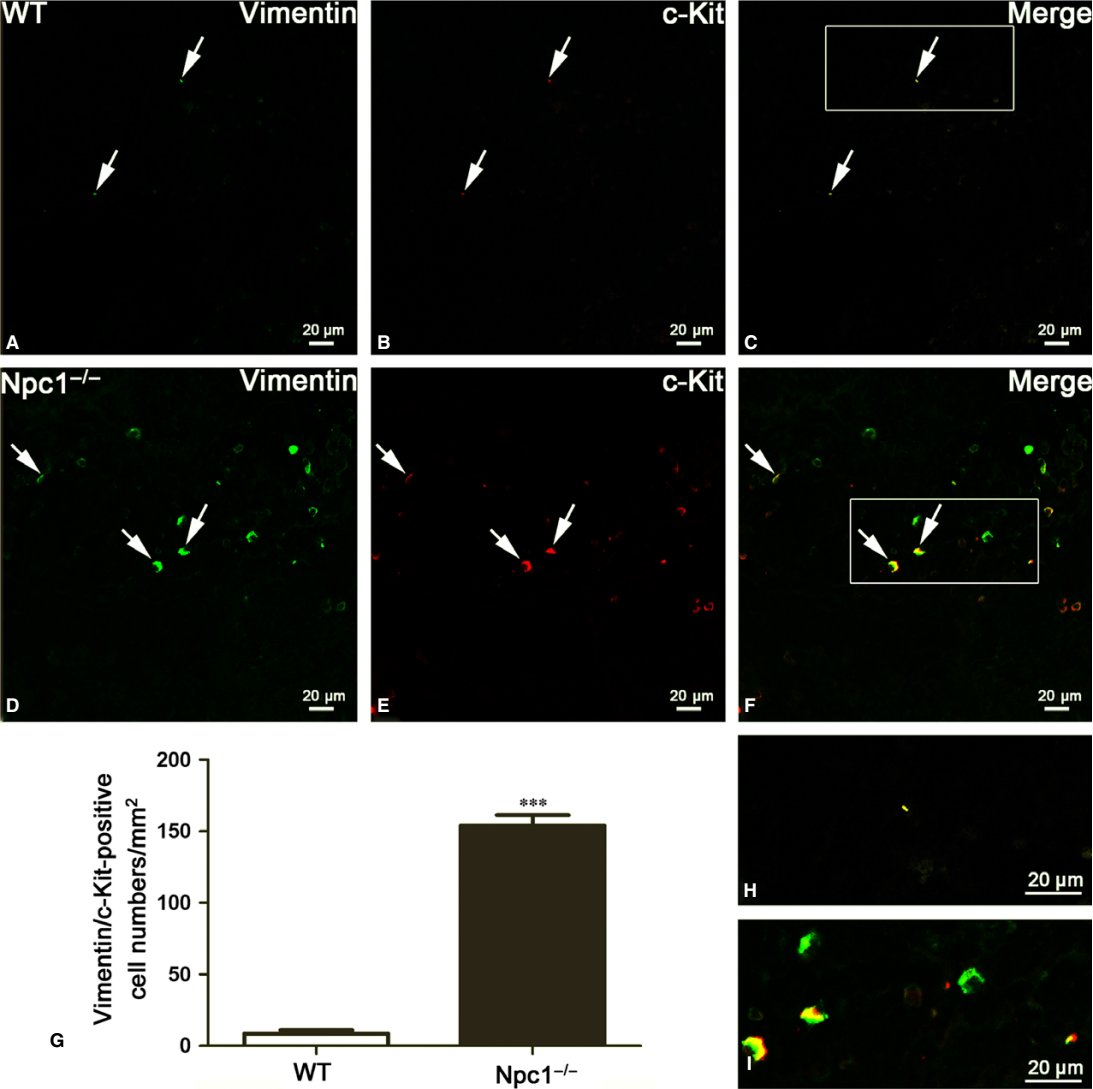
Splenic telocytes (TCs) are identified by Vimentin/c‐Kit double‐immunofluorescent staining from confocal laser scanning microscopy. Vimentin and c‐Kit double immunostaining show Vimentin (green in **A** and **D**), c‐Kit (red in **B** and **E**) and their coexpression merge (yellow in **C** and **F**) in telocytes of WT and Npc1^−/−^ mice. TCs in the spleen of Npc1^−/−^ mice are increased as determined by double‐immunofluorescence labelling for Vimentin/c‐Kit (**G**), **H** and **I** are the partial amplification from boxed area in **C** and **F**, respectively. Scale bar = 20 μm labelled in each panel, *n* = 4. ***P < 0.001.

Although transmission electron microscopy is a golden standard for TC identification, double‐positive immunostaining is now the most common method to analyse TCs. Here, three distinct double‐immunostaining combinations for c‐Kit/CD34 (Fig. 3), Vimentin/CD34 (Fig. 4) and Vimentin/c‐Kit (Fig. 5) were performed to determine the alteration of TCs in spleen of Npc1^−/−^ mice 18, 19, 20, 22, 23.

TCs were originally considered c‐Kit‐positive based on their resemblance to interstitial cells of Cajal (ICC) 30, 35. Some other studies have noted that TCs could be CD34‐positive 36. Hitherto, double‐positive immunostaining with CD34/c‐Kit (mainly for cell body) was considered useful markers for TCs. Double immunohistochemistry against c‐Kit and CD34 for TCs reveals the large majority of c‐Kit‐positive labelling (green colour in Fig. 3D) in the Npc1^−/−^ spleen, which coexpress CD34 (red and yellow colour in Fig. 3E and F), but it was rarely in the wild‐type control (Fig. 3A–C). The quantitative analysis showed that TCs labelled by c‐Kit/CD34 in spleen of Npc1^−/−^ mice were dramatically increased with a density of 165–166 cells/mm^2^, compared to the control 8–10 cells/mm^2^ (Fig. 3G).

As reported, Vimentin/CD34‐positive cells are also considered as TCs (Fig. 4) 19, 20. In the wild‐type control, seldom is double‐labelled by Vimentin and CD34 (Fig. 4A–C), whereas large Vimentin/CD34‐positive cells are observed in the spleen of Npc1^−/−^ mice (Fig. 4D–F). Quantitative analysis based on Vimentin and CD34 double labelling showed a significant elevation in the number of splenic TCs in Npc1^−/−^ mice (Fig. 4G). The density of these cells calculated on images of immunostaining was 8–10 and 118–119 cells/mm^2^, respectively (Fig. 4G).

Similar results occurred in Vimentin/c‐Kit double‐immunostaining for splenic TCs (Fig. 5). Immunohistochemistry for Vimentin and c‐Kit revealed multiple double‐positive cells in the spleen of Npc1^−/−^ mice (Fig. 5D–F), but in the wild‐type control, it is rare (Fig. 5A–C). The appearance of these Vimentin/c‐Kit‐positive cells is indicative of TCs (Fig. 5C and F). The Vimentin/c‐Kit‐positive TCs are strikingly elevated in the spleen of Npc1^−/−^ mice with a density of 154–156 cells/mm^2^, compared to the control 8–10 cells/mm^2^ (Fig. 5G). Taken together, these results from transmission electron microscopy examination (Fig. 2) and three distinct double immunostainings (Figs. 3, 4, 5) demonstrated that the numbers of splenic TCs are dramatically increased in Npc1^−/−^ mice.

### Enhanced pluripotency of stem cells in the spleen of Npc1^−/−^ mice

**Figure 6:**
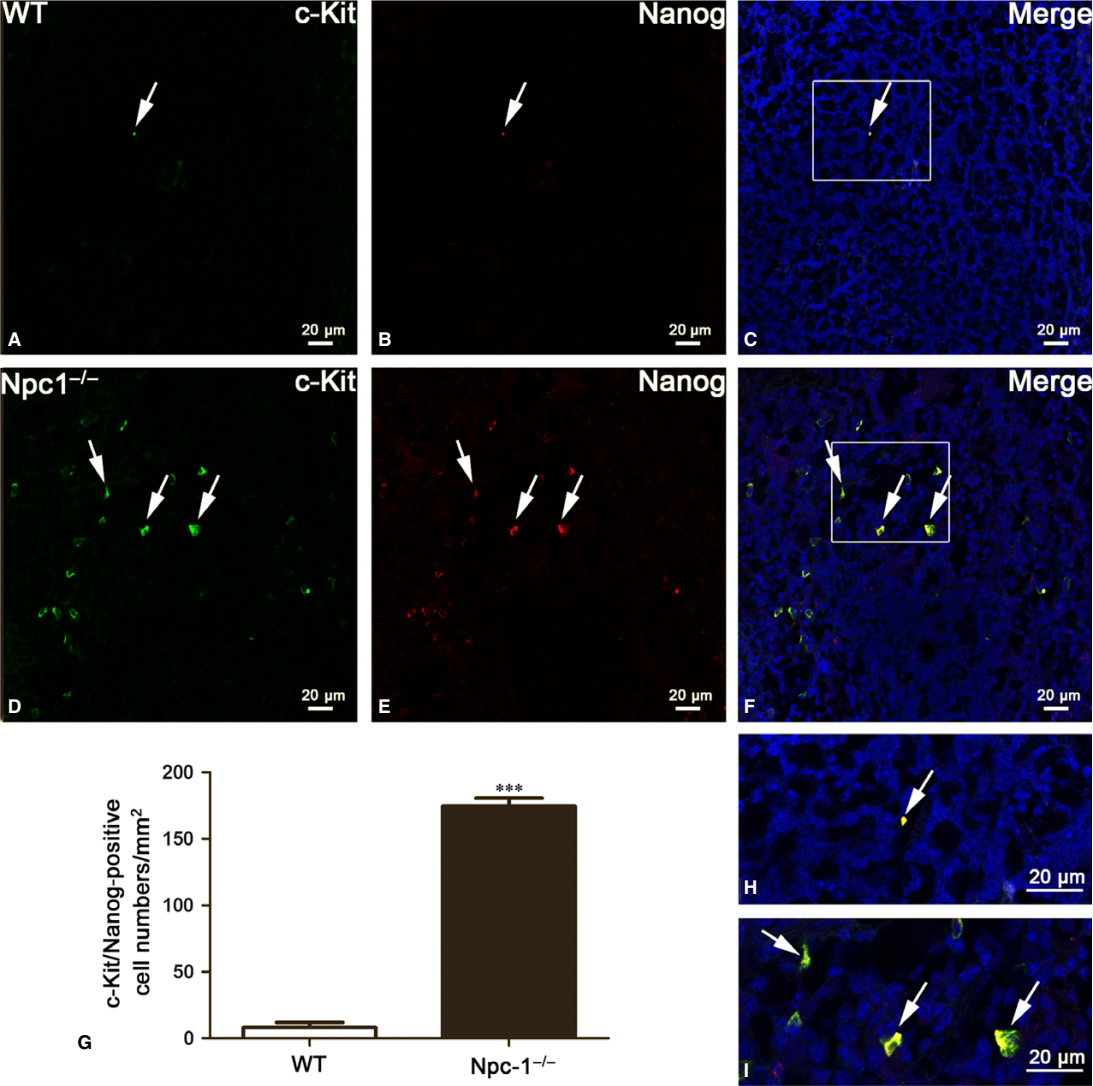
Stem cells are increased in the spleen of Npc1^−/−^ mice. Nanog and c‐Kit double immunostaining show c‐Kit (green in **A** and **D**), Nanog (red in **B** and **E**) and their coexpression merge (yellow in **C** and **F**) in telocytes of WT and Npc1^−/−^ mice. Stem cells in the spleen of Npc1^−/−^ mice are increased as labelled by c‐Kit/Nanog (**G**). **H** and **I** are the partial amplification from boxed area in **C** and **F**, respectively, scale bar = 20 μm labelled in each panel, *n* = 4. ***P < 0.001.

c‐Kit is also an important cell surface marker used to identify certain types of hematopoietic progenitors 37. Meanwhile, Nanog is a transcription factor in embryonic stem cells and is thought to be a key factor maintaining pluripotency 38. Here, we used c‐Kit/Nanog double labelling to detect the hematopoietic stem cells in spleen of Npc1^−/−^ mice (Fig. 6). The results demonstrated that the number of c‐Kit/Nanog double‐labelled cells was rarely found in the control mice (Fig. 6B and C), but frequently in the in the Npc1^−/−^ spleen (Fig. 6E and F). In agreement with the results of TCs, Fig. 6G indicated that hematopoietic stem cell identified by c‐Kit and Nanog was also augmented in Npc1^−/−^ mice.

**Figure 7:**
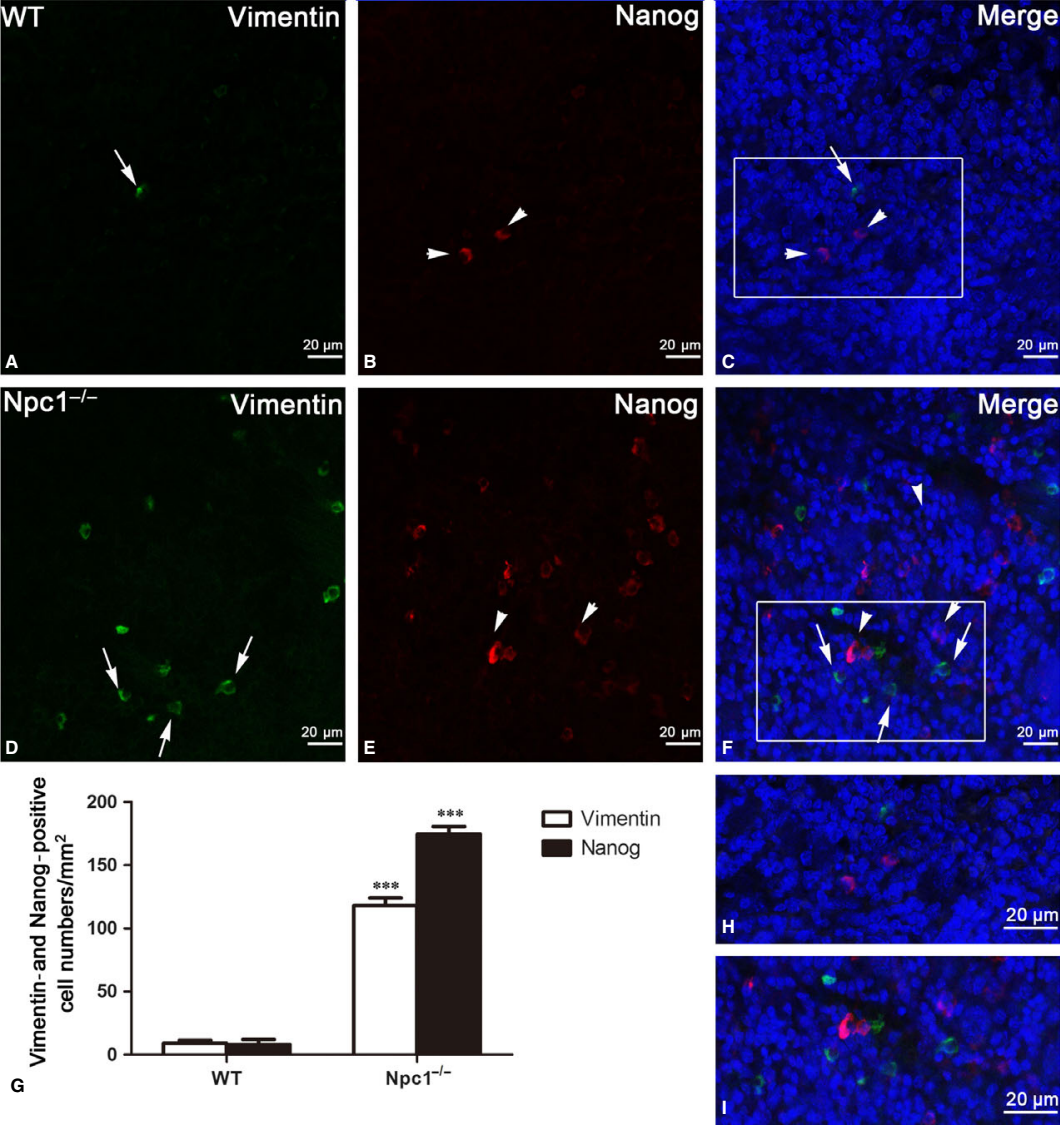
Vimentin/Nanog double‐immunofluorescence labelling shows the costaining of telocytes(TCs) and hematopoietic stem cells (HSCs). Laser scanning confocol microscopy: Vimentin and Nanog double immunostaining show Vimentin (green in **A** and **D**), Nanog (red in **B** and **E**) and their colocalization (**C** and **F**) in telocytes (green) and hematopoietic stem cells (red) of WT and Npc1^−/−^ mice. TCs and HSCs in the spleen of Npc1^−/−^ mice are increased as determined by double‐immunofluorescence labelling for Vimentin and Nanog (**G**). **H** and **I** are the partial amplification from boxed area in **C** and **F**, respectively. Scale bar = 20 μm labelled in each panel, *n* = 4. ***P < 0.001.

To further investigate the link between the increases of TCs and hematopoietic stem cells, double immunostaining against Vimentin and Nanog was performed (Fig. 7). Immunofluorescent staining against Vimentin and Nanog shows that hematopoietic stem cells (red colour in Fig. 7B and E) are peripherally distributed in the region of TCs (green colour in Fig. 7A and D), as shown in the Figure 7C and F. Interestingly, the amount of hematopoietic stem cell in the spleen of Npc1^−/−^ is dramatically elevated along with the increase in TCs, compared to the control group (Fig. 7G).

### Increased activities of Macrophages in the spleen of Npc1^−/−^ mice

**Figure 8:**
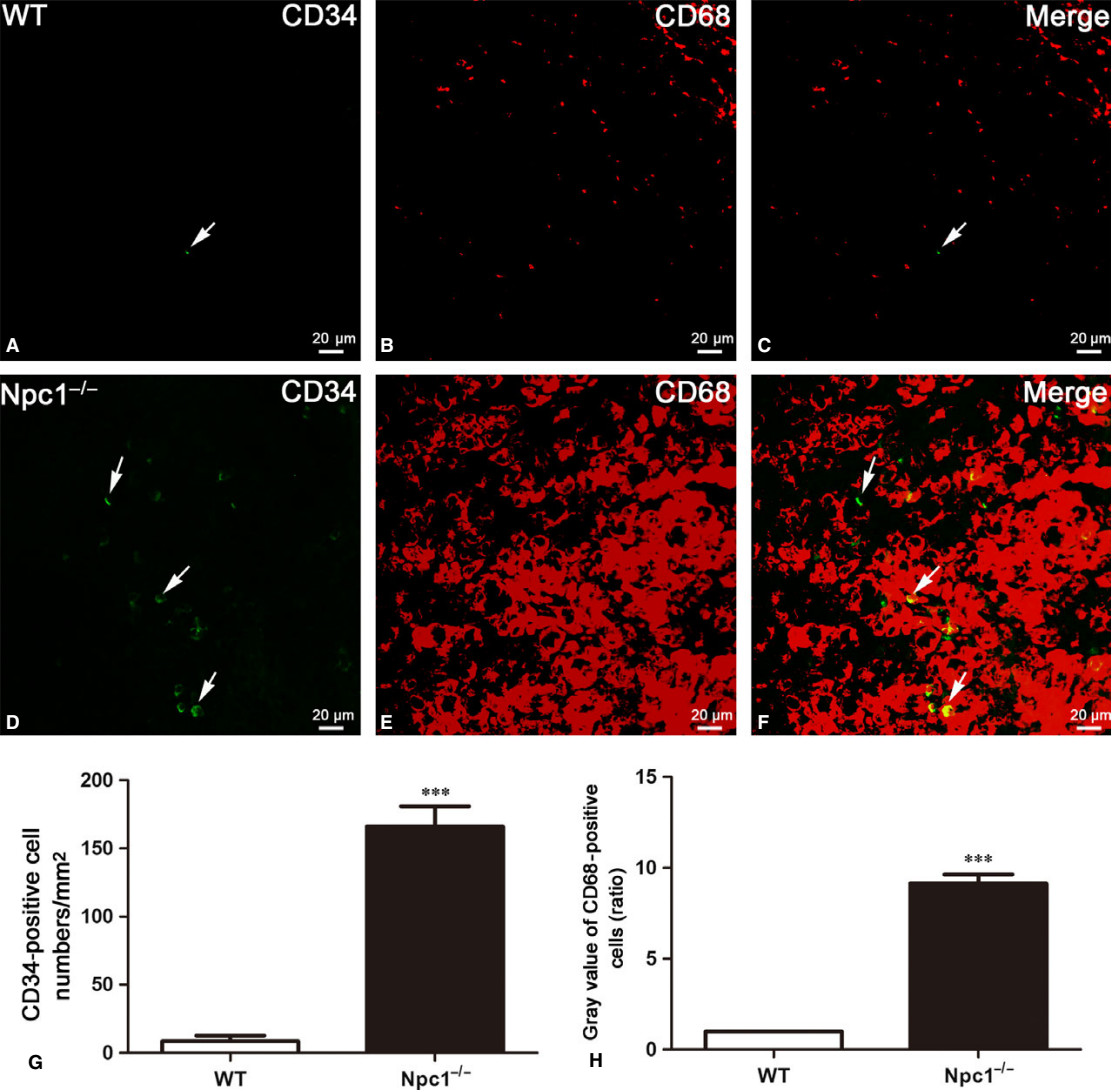
CD34/CD68 double‐immunofluorescence labelling shows the costaining of telocytes(TCs) and macrophages. CD34 and CD68 double immunostaining shows CD34 (green in **A** and **D**), CD68 (red in **B** and **E**) and their colocalization (**C** and **F**) in telocytes (green) and macrophages (red) of WT and Npc1^−/−^ mice. TCs and macrophages in the spleen of Npc1^−/−^ mice are increased as determined by double‐immunofluorescence labelling for CD34 (**G**) and CD68 (**H**). The grey value of CD68^+^ cells in the control spleen was considered as 1, and the ratio showed the relative amount of CD68^+^ cells in Npc1^−/−^ mouse spleen. Scale bar = 20 μm labelled in each panel, *n* = 3. ***P < 0.001.

CD68, which is homologous to the mouse antigen macrosialin 39,belongs to a family of acidic, highly glycosylated lysosomal glycoproteins (LGPs) 40. It is found on the surface of macrophages, monocytes, neutrophils, basophils and large lymphocytes, commonly regarded as a macrophage marker 40, 41, 42, 43. To investigate the changes of macrophages and the connection between TCs and macrophages in spleen of Npc1^−/−^ mice, immunostaining was performed using antibody against CD34 and CD68 for TCs (green colour in Fig. 8A and D) and macrophages (red colour in Fig. 8B and E), respectively, as shown in Figure 8. Only a few CD68‐positive cells were detected in the wild‐type mouse (Fig. 8B), but the number of CD68‐positive cells found in the Npc1^−/−^ mouse was significantly higher (Fig. 8E). Analysis of grey value for Figure 8B and E showed that the relative grey value of Npc1^−/−^ is much higher than that of WT, almost up to tenfold, which demonstrated the considerable increase in the number of macrophages in Npc1^−/−^ mice (Fig. 8H). Furthermore, those macrophages had a larger cell body (Fig. 8B) compared to the wild‐type ones (Fig. 8E), suggesting an increase in macrophage activity. In addition, immunohistochemistry for Vimentin and c‐Kit revealed that macrophages in the spleen of Npc1^−/−^ mice are considerably augmented with the increase in TCs (Fig. 8G and H).

## Discussion

Increasing evidence has demonstrated the potential roles of TCs in regenerative medicine 44, 45, 46. This study provides a novel finding that splenic TCs are increased in Npc1^−/−^ mice. Moreover, our data presented here suggest that splenic TCs might contribute to alleviate the progress of splenic malfunction.

The spleen is a centre of activity of the mononuclear phagocyte system and can be considered analogous to a large lymph node 47. It is found recently that the red pulp of the spleen forms a reservoir that contains over half of the body's monocytes 48. These monocytes, upon moving to injured tissue (such as the heart after myocardial infarction), turn into dendritic cells and macrophages while promoting tissue healing 48, 49. In NCTR‐BALB/c mice, the spleen was extensively infiltrated with large foam cells, most of which were presumably derived from free or fixed macrophages 50. Besides, it is observed in our study that the size of spleen in Npc1^−/−^ mice is enlarged, one of hallmarks of Npc1, which is consistent with previous reports 14.

TCs are presumably considered as nurse cells for progenitors. It has been reported that cardiac TCs could nurse putative cardiomyocyte precursors to differentiate and integrate into heart architecture 20, 45, 51. In mouse liver, TCs might influence proliferation of hepatocytes and/or the activation of stem/progenitor cells 44. Chang *et al*. 19 also proposed that splenic TCs could participate in the formation of splenic hematopoietic niche and play a critical role in intercellular communication and regulation. In our study, the splenic TCs have been detected in WT and Npc1^−/−^ mice with the characteristic morphology: an oval or triangular‐shaped cell body and two or more long and thin Tps (Fig. 2). Interestingly, we demonstrated in our study that the numbers of TCs are strikingly elevated in the enlarged spleen of Npc1^−/−^ mice, based on transmission electron microscopy examination and three distinct double immunostainings for c‐Kit/CD34, Vimentin/CD34 and Vimentin/c‐Kit. This phenomenon undoubtedly pointed out that TCs could play a vital role in the progress of splenic malfunction in Npc1^−/−^ mice. In addition, c‐Kit/Nanog and Vimentin/Nanog double labelling indicated a significant increase in the number of hematopoietic stem cells along with the alteration of splenic TCs in Npc1^−/−^ mice. These data imply that splenic TCs might play an important role in nursing or recruiting stem cells. It is worth of noting that, in various organs, Tps could establish close synapse‐like contacts with immunoreactive cells 28. Moreover, Zheng *et al*. 52 reported that TCs may be responsible for the orientation and recruitment of inflammatory cells from circulation into the tissue during the infectious process. What coincidence is that our data show that the elevation of splenic TCs in Npc1^−/−^ mice was followed by a dramatic increase in macrophages, indicating that macrophages were recruited by TCs during the pathologic process of Npc1. Together, these results suggest that splenic TCs might recruit hematopoietic stem cells and macrophages with response to excessive foam cells resulted from a lysosome storage syndrome in Npc1^−/−^ mice.

In conclusion, splenic TCs might act as a defender for spleen through recruiting hematopoietic stem cells and macrophages to alleviate the progress of splenic malfunction in Npc1^−/−^ mice. Concerning the mechanism about how TCs influence the pluripotency of hematopoietic stem cells and activities of macrophages in the spleen of Npc1^−/−^ mice, it is still unclear and that also happens to be what we are going to investigate subsequently.

## Conflict of interest

The authors declare there are no conflicts of interest.
